# Antibacterial activities of the extracts, fractions and compounds from *Dioscorea bulbifera*

**DOI:** 10.1186/1472-6882-12-228

**Published:** 2012-11-23

**Authors:** Victor Kuete, Rémy BetrandTeponno, Armelle Tsafack Mbaveng, Léon Azefack Tapondjou, Jacobus J Marion Meyer, Luciano Barboni, Namrita Lall

**Affiliations:** 1 Department of Biochemistry, Faculty of Science, University of Dschang, P.O. Box 67, Dschang, Cameroon; 2 Department of Chemistry, Faculty of Science, University of Dschang, Dschang, Cameroon; 3 Department of Plant Science, Faculty of Agricultural and Biological Science, Pretoria, 0002, South Africa; 4 School of Science and Technology, Chemistry Division, University of Camerino, Via S. Agostino 1, I-62032, Camerino, Italy

**Keywords:** Diterpenoids, Antimycobacterial, Antibacterial, *Dioscorea bulbifera*, *Dioscoreaceae*

## Abstract

**Background:**

*Dioscorea bulbifera* is an African medicinal plant used to treat microbial infections. In the present study, the methanol extract, fractions (DBB1 and DBB2) and six compounds isolated from the bulbils of *D*. *bulbifera*, namely bafoudiosbulbins A (**1**), B (**2**), C (**3**), F (**4**), G (**5**) and 2,7-dihydroxy-4-methoxyphenanthrene (**6**), were tested for their antimicrobial activities against Mycobacteria and Gram-negative bacteria involving multidrug resistant (MDR) phenotypes expressing active efflux pumps.

**Methods:**

The microplate alamar blue assay (MABA) and the broth microdilution methods were used to determine the minimal inhibitory concentration (MIC) and minimal bactericidal concentration (MBC) of the above samples.

**Results:**

The results of the MIC determinations indicated that when tested alone, the crude extract, fractions DBB1 and DBB2 as well as compounds **2** to **5** were able to prevent the growth of all the fifteen studied microorganisms, within the concentration range of 8 to 256 μg/mL. The lowest MIC value for the methanol extract and fractions (16 μg/mL) was obtained with DBB1 and DBB2 on *E*, *coli* AG100A and DBB2 on *Mycobacterium tuberculosis* MTCS2. The lowest value for individual compounds (8 μg/mL) was recorded with compound **3** on *M*. *smegmatis* and *M*. *tuberculosis* ATCC and MTCS2 strains respectively. The activity of the samples on many MDR bacteria such as *Enterobacter aerogenes* EA289, CM64, *Klebsiella pneumoniae* KP63 and *Pseudomonas aeruginosa* PA124 was better than that of chloramphenicol. When tested in the presence of the efflux pump inhibitor against MDR Gram-negative bacteria, the activity of most of the samples increased. MBC values not greater than 512 μg/mL were recorded on all studied microorganisms with fraction DBB2 and compounds **2** to **5**.

**Conclusions:**

The overall results of the present investigation provided evidence that the crude extract *D*. *bulbifera* as well as some of the compounds and mostly compounds **3** could be considered as potential antimicrobial drugs to fight against MDR bacteria.

## Background

The continuous emergence of Gram-negative MDR bacteria drastically reduces the efficacy of our antibiotic armory and, consequently, increases the frequency of therapeutic failure [1]. On the other hand, the World Health Organization (WHO) estimates that there are nine million cases of tuberculosis (TB) currently, with 1.3 million reported deaths every year, 55 and 30% of the TB burden being shared by Asian and African countries respectively [2]. Approximately 60% of world’s population still relies on medicinal plants for their primary healthcare. Medicinal plants have been used as a source of remedies since ancient times in Africa. *Dioscorea bulbifera* L. var *sativa* (Dioscoreaceae) is an African medicinal plant used to treat microbial infections and pig cysticercosis by the native people of western highlands of Cameroon. The plant is also used as a folk remedy to treat conjunctivitis, diarrhea and dysentery, among other ailments [3]. Previous phytochemical study on this medicinal plant led to the isolation and structural elucidation of seven new clerodane diterpenoids namely Bafoudiosbulbins A-G [4-6]. Furthermore, the extracts and Bafoudiosbulbins A and B were shown to possess anti-*Salmonella* activity [4]. In the present study, the bioguided fractionation was undertaken in order to deeply evaluate the antimicrobial activity of *D*. *bulbifera*.

## Methods

### Plant material

The bulbils of *D*. *bulbifera* L. var *sativa* were collected in Bafou village near Dschang (West region of Cameroon) in February 2007. The plant was identified at the National Herbarium (Yaoundé, Cameroon) where a voucher specimen was deposited under the reference number 22211/SRF/CAM.

### Extraction and isolation

The air-dried bulbils of *D*. *bulbifera* L. var *sativa* (2 kg) were pulverized and extracted three times (each time for 24 h) with MeOH. The methanol extract was concentrated under reduced pressure to yield a dark residue (DBB; 90 g). Part of this (80 g) was suspended in water (150 mL) and submitted to successive partition with Ethyl acetate (EtOAc) and *n*-butanol. The EtOAc and *n*-butanol layers were concentrated to dryness under reduced pressure to afford 35 g and 23 g of extracts respectively. The *n*-butanol extract showed no antimicrobial activity contrary to the EtOAc extract (Tables 1 and 2). Part of the EtOAc extract (DBB1; 28 g) was subjected to column chromatography over silica gel 60 Merck [0.040–0.063 mm; 56 g] using hexane-EtOAc with increasing polarity as eluents to yield five main sub-fractions named A-E. They were then screened for their antimicrobial activities and sub-Fraction C (DBB2) eluted with Hexane-EtOAc 4–6 to 3–7 was the only active sample. DBB2 (m = 4.8 g) was then fractionated and purified using column chromatography over silica gel and sephadex LH-20 to yield compound **1** {White needles; [α]_D_^21^ = − 64.6 (c = 0.025, DMSO); 60 mg; Rf = 0.78, CH_2_Cl_2_-MeOH 95–5; C_21_H_22_O_8_*m*/*z* 403 [M+H]^+^; m.p. = 252-253°C}, compound **2** {White needles; [α]_D_^21^ = +52 (c = 0.010, pyridine); 71 mg; Rf = 0.44, CH_2_Cl_2_-MeOH 95–5; C_20_H_20_O_8_, *m*/*z* 387 [M−H]^−^; m.p. = 312-313°C}, compound **3** {White gum; [α]_D_^21^ = +56.2° (c = 0.8, CH_2_Cl_2_); 32 mg; Rf = 0.37, CH_2_Cl_2_-MeOH 95–5; C_21_H_22_O_7_, 358 [M−CO]^+^}, compound **4** {White needles; [α]_D_^21^ = − 5^o^ (c = 0.6, CH_2_Cl_2_); 58 mg; Rf = 0.56, CH_2_Cl_2_-MeOH 95–5; C_21_H_24_O_8_; *m*/*z* 439 [M+Cl]; m.p. = 265-266°C}, compound **5** {White crystals; [α]_D_^20^ = − 46^o^ (c = 0.9, CD_3_OD); 64 mg; Rf = 0.41, CH_2_Cl_2_-MeOH 95–5; C_23_H_26_O_10_; *m*/*z* 485 [M+Na]^+^; m.p. = 199-200°C}, compound **6** {Yellow amorphous powder; 28 mg; Rf = 0.48, CH_2_Cl_2_-MeOH 95–5; C_15_H_12_O_3_, *m*/*z* 241 [M+H]^+^}.

**Table 1:** **MICs of the extract**, **fractions**, **compounds from *Dioscorea bulbifera *and chloramphenicol on documented strains and clinical MDR isolates**

**Samples**^**a**^	**Microorganisms and MIC(μg**/**mL)without and in the presence of PAßN(in parenthesis)**^**b**^														
	***E.coli***			***E.aerogenes***			***K.pneumoniae***			***P.aeruginosa***		***M.smegmatis***	***M.tuberculosis***		
	**ATCC8739**	**AG100A**	**AG102**	**ATCC13048**	**EA289**	**EA**-**CM64**	**ATCC11296**	**KP55**	**KP63**	**PA01**	**PA124**	***ATCC 700084***	***ATCC 27294***	***MTCS1***	***MTCS2***
DBB	64 (64)	64 (32)	128 (128)	64 (64)	256 (128)	256 (256)	64 (64)	128 (128)	128 (128)	128 (128)	128 (128)	64	64	256	64
DBB1	32 (32)	16 (8)	128 (128)	32 (32)	64 (64)	64 (64)	32 (32)	64 (64)	64 (64)	64 (64)	64 (64)	3x2	64	256	32
DBB2	64 (64)	16 (8)	64 (64)	32 (16)	64 (64)	64 (64)	64 (64)	64 (64)	32 (16)	32 (16)	64 (64)	32	32	64	16
**1**	256 (64)	128 (32)	-	256 (128)	- (256)	-	256 (64)	512 (128)	- (256)	- (512)	-	-	nd	nd	nd
**2**	64 (32)	64 (64)	64 (32)	128 (64)	64 (64)	64 (64)	64 (32)	128 (64)	64 (32)	128 (64)	128 (64)	32	32	32	16
**3**	64 (16)	16 (8)	64 (16)	64 (16)	64 (16)	64 (16)	64 (32)	64 (16)	64 (32)	128 (32)	128 (32)	8	8	32	8
**4**	64 (64)	64 (32)	64 (64)	128 (32)	64 (32)	128 (64)	64 (64)	64 (64)	64 (32)	128 (64)	128 (64)	32	64	64	32
**5**	64 (64)	64 (32)	128 (64)	128 (64)	128 (32)	256 (64)	128 (128)	128 (64)	256 (64)	256 (64)	128 (32)	64	32	64	32
**6**	256 (256)	256 (256)	- (512)	- (512)	- (256)	-	256 (128)	- (256)	- (256)	- (256)	- (512)	-	nd	nd	nd
RA	4 (1)	1 (0.5)	16 (1)	4 (1)	512 (128)	256 (8)	2 (2)	32 (4)	512 (128)	128 (8)	256 (8)	0.5	0.5	64	2

^a^Tested samples [DBB: methanol extract from the bulbils of *D*. *bulbifera*; bafoudiosbulbins A (**1**), B (**2**), C (**3**), F (**4**), G (**5**), 2,7-dihydroxy-4-methoxyphenanthrene (**6**); EtOAc fraction (DBB1) and EtOAc sub-fraction C (DBB2); RA : reference antibiotics were chloramphenicol for bacteria, ciprofloxacin for *M*. *smegmatis*, isoniazid for *M*.*tuberculosis*; (−): MIC > 512 μg/mL; ^*b*^The drugs and compounds were tested in the absence or in the presence (values in braket) of PAßN at a final concentration of 20 μg/mL, as described previously [7]. At this concentration, no intrinsic effect against the various bacterial strains (included as internal controls in each assay without antibiotic) was observed.

**Table 2:** **MBCs of the extract**, **fractions**, **compounds from *Dioscorea bulbifera *and chloramphenicol on documented strains and clinical MDR isolates**

**Samples**	**Microorganisms and MBC(μg**/mL)without and in the presence of PAßN(in parenthesis)^*^														
	***E.coli***			***E.aerogenes***			***K.pneumoniae***			***P.aeruginosa***		***M.smegmatis***	***M.tuberculosis***		
	**ATCC8739**	**AG100A**	**AG102**	**ATCC13048**	**EA289**	**EA**-**CM64**	**ATCC11296**	**KP55**	**KP63**	**PA01**	**PA124**	***ATCC 700084***	***ATCC 27294***	***MTCS1***	***MTCS2***
DBB	128 (128)	128 (64)	256 (256)	128 (128)	512 (512)	- (512)	256 (256)	256 (256)	256 (256)	256 (256)	256 (256)	128	128	-	128
DBB1	128 (64)	32 (16)	512 (256)	64 (64)	128 (128)	256 (128)	64 (64)	128 (128)	256 (128)	128 (128)	128 (128)	64	128	-	64
DBB2	256 (128)	32 (16)	256 (128)	64 (32)	128 (128)	256 (128)	128 (128)	128 (128)	64 (64)	64 (64)	256 (128)	128	128	128	64
**1**	- (256)	256 (128)	nd	- (256)	nd	nd	- (128)	- (256)	nd	nd	nd	-	nd	nd	nd
**2**	128 (128)	128 (64)	128 (128)	256 (128)	128 (128)	128 (128)	128 (128)	256 (128)	128 (128)	512 (128)	256 (128)	64	128	128	64
**3**	128 (32)	128 (32)	256 (32)	128 (32)	256 (32)	128 (32)	128 (64)	128 (64)	256 (64)	256 (64)	256 (64)	8	16	64	16
**4**	128 (128)	128 (64)	256 (128)	256 (128)	128 (64)	512 (128)	256 (128)	256 (128)	128 (128)	256 (128)	256 (128)	64	128	128	128
**5**	128 (128)	128 (128)	256 (128)	256 (128)	256 (128)	- (128)	512 (256)	512 (128)	- (128)	512 (256)	512 (128)	256	128	256	128
**6**	-	- (512)	-	nd (−)	nd (256)	nd	- (512)	nd (256)	nd (256)	nd (256)	nd (512)	-	nd	nd	nd
RA	8 (2)	4 (1)	32 (2)	8 (2)	- (256)	512 (16)	4 (4)	64 (8)	- (256)	256 (16)	512 (16)	1	1	128	4

^a^Tested samples [DBB: methanol extract from the bulbils of *D*. *bulbifera*; bafoudiosbulbins A (**1**), B (**2**), C (**3**), F (**4**), G (**5**), 2,7-dihydroxy-4-methoxyphenandhrene (**6**); EtOAc fraction (DBB1) and EtOAc sub-fraction C (DBB2); RA : reference andibiotics were chloramphenicol for bacteria, ciprofloxacin for *M*. *smegmatis*, isoniazid for *M*.*tuberculosis*; (−): MIC > 512 μg/mL; ^*b*^The drugs and compounds were tested in the absence or in the presence (values in braket) of PAßN at a final concendration of 20 μg/mL, as described previously [7]. At this concendration, no indrinsic effect against the various bacterial strains (included as indernal condrols in each assay without andibiotic) was observed; (nd): not determined.

### General procedure

Melting points were determined using the GallenkampMelting Point Apparatus. Optical rotations were measured on a Perkin–Elmer 241 Polarimeter, IR spectra were measured as a film on a KBr pellet using a FTIR-8400S Shimadzu spectrometer. ESIMS was carried out on a Hewlett Packard HP-1100 series LC–MSD system and on the mass spectrometer Brucker FTMS4.7T, BIOAPEXII. ^1^H NMR spectra were recorded in deuterated solvents (DMSO and C_5_H_5_N) on a on a Varian Mercury Plus Spectrometer at 400 MHz while ^13^C NMR spectra were recorded in the same solvents and the same apparatus at 100 MHz. All chemical shifts (*δ*) are given in ppm units with reference to tetramethylsilane (TMS) as internal standard and the coupling constants (*J*) are in Hz. Column chromatography was performed using silica gel 60 Merck (0.040–0.063 mm) and sephadex LH-20. TLC were carried out on precoated Kieselgel 60 F254 (Merck) plates developed with hexane:AcOEt (7:3) and AcOEt:MeOH (98:2). TLC plates were viewed with an ultraviolet lamp MULTIBAND UV-254/365 nm for fluorescent spots. They were also visualized by spraying with 50% H_2_SO_4_ and heating for 10 min at 110°C.

### Antimicrobial assays

#### Chemicals for antimicrobial assay

Chloramphenicol ≥ 98% (Sigma-Aldrich, St. Quentin Fallavier, France) was used as reference antibiotics (RA) against Gram-negative bacteria. *p*-Iodonitrotetrazolium chloride ≥ 97% (INT, Sigma-Aldrich) was used as microbial growth indicator [8,9]. Ciprofloxacin ≥ 98% and isoniazid ≥ 99% (INH) (Sigma) were used as reference antibiotics (RA) for *M*. *smegmatis* and *M*. *tuberculosis* respectively. Phenylalanine arginine β-naphthylamide ≥ 98% (PAßN, Sigma-Aldrich) was used as microbial growth indicator and efflux pumps inhibitor.

#### Microbial strains and culture media

The studied microorganisms included strains of *Pseudomonas aeruginosa*, *Klebsiella pneumoniae*, *Enterobacter aerogenes*, *Escherichia coli*, four Mycobacteria namely *M*. *smegmatis*, drug-susceptible strain of *M*. *tuberculosis* H37Rv obtained from the American Type Culture Collection, and two clinical strains of *M*. *tuberculosis* MTCS1, MTCS2. *M*. *smegmatis* was cultured on Middlebrook 7H11 agar and allowed to grow for 24 h. *M*. *tuberculosis* was plated on Löwenstein–Jensen medium and allowed to grow for 3–4 weeks at 37°C. Middlebrook 7H9 broth was used to determine the MIC and MBC values of the test samples on *M*. *smegmatis* and *M*. *tuberculosis*. Nutrient agar was used for the activation of Gram-negative bacteria [10]. The clinical features of the Gram-negative bacteria are available as Additional file 1.

#### INT colorimetric assay for MIC and MBC determinations

The MIC determinations on *M*. *smegmatis* and Gram-negative bacteria were conducted using rapid INT colorimetric assay according to described methods [8,9] with some modifications. The test samples and RA were first of all dissolved in DMSO/Mueller Hinton Broth (MHB) or DMSO/7H9 broth. The final concentration of DMSO was lower than 2.5% and does not affect the microbial growth [11]. The solution obtained was then added to 7H9 broth (*M*. *smegmatis*) or Mueller Hinton Broth (Gram-negative organisms), and serially diluted two fold (in a 96- wells microplate). One hundred microlitre (100 μL) of inoculum 1.5 x 10^6^ CFU/mL prepared in appropriate broth was then added [12]. The plates were covered with a sterile plate sealer, then agitated to mix the contents of the wells using a plate shaker and incubated at 37°C for 18 h. The assay was repeated thrice. Wells containing adequate broth, 100 μL of inoculum and DMSO to a final concentration of 2.5% served as negative control. The MIC of samples was detected after 18 h incubation at 37°C, following addition (40 μL) of 0.2 mg/mL *p*-iodonitrotetrazolium chloride (INT) and incubation at 37°C for 30 min. Viable bacteria reduced the yellow dye to a pink. MIC was defined as the sample concentration that prevented the color change of the medium and exhibited complete inhibition of microbial growth [8,9]. The MBC was determined by adding 50 μL aliquots of the preparations, which did not show any growth after incubation during MIC assays, to 150 μL of adequate broth. These preparations were incubated at 37°C for 48 h. The MBC was regarded as the lowest concentration of extract, which did not produce a color change after addition of INT as mentioned above [11,13].

Samples were also tested in the presence of PAßN at 30 μg/mL final concentration [7] and the MIC was determined as above.

#### Time-kill dynamic curves against E. coli ATCC8739

Time-kill dynamic assay was performed using broth microdilution method as previously described [14] with minor modifications. The final concentration of suspension of the *E*. *coli* ATCC8739 strain was adjusted to 10^6^ CFU/mL. The crude extract, fraction DBB2 and compound **3** were used in the time-kill dynamic experiment. Cells treated with concentrations corresponding to ½ MIC; MIC and MBC of each sample were incubated at 37°C for 0, 30, 60, 120, 240, 480, and 960 min. The final concentration of DMSO was 2.5%. A control sample was made using DMSO 2.5% and the inoculum. At each incubation time point, liquids (50 μl) were removed from the test solution for ten-fold serial dilution. Thereafter, a 25 μl liquid from each dilution was spread on the surface of the MHA plates and incubated at 37°C for 24 h, and the number of CFU/mL was counted. Experiments were carried out in triplicate. Time-kill curves were constructed by plotting the number of CFU/mL against time (min).

#### Microplate Alamar Blue assay against M. tuberculosis

The activity of all samples against *M*. *tuberculosis* strains was tested using the MABA [15]. Briefly, each of the above *M*. *tuberculosis* strains was cultured at 37°C in Middlebrook 7H9 broth supplemented with 0.2% glycerol and 10% Oleic Acid–Albumin–Dextrose–Catalase (Sigma) until logarithmic growth was reached. About 6x10^6^ CFU/mL inoculum of *M*. *tuberculosis* was then added to the two fold serially diluted samples. The final concentration of DMSO in all assays was 2.5% or less and this dilution also served as solvent control. The samples were assayed in triplicate. All tests were carried out in sterile flat bottom 96-well microplates. Each microplate was incubated for 5 days at 37°C in a sealed plastic CO_2_-permeable bag. After 5 days of incubation, 32 μL of a mixture of freshly prepared Alamar Blue solution and 20% sterile Tween-80 (Sigma) 1:1 v/v were added to one growth-control well. The microplates were incubated again at 37°C for 24 h. If a color shift from blue to pink was observed in the growth-control sample, 32 μL of alamar blue solution was added to each of the remaining wells, and the microplate was further incubated for 24 h. A well-defined pink color was interpreted as positive bacterial growth, whereas a blue color indicated an absence of growth. The MIC corresponded to the greatest dilution of sample extract in which the color shift from blue to pink was not observed.

Samples with recorded MIC values following MABA were assayed for their mycobactericidal effect [15]. Briefly, 5 μL of the undeveloped mycobacterial suspensions were transferred from the former to a new microplate that contained 195 μL of fresh culture medium per well. Three wells were inoculated with 100 μL of fresh inoculum as for MABA and three more wells were incubated with 200 μL of culture medium only, as negative controls. The microplates were incubated and developed with alamar blue as for MABA. The MBC corresponded to the minimum sample concentration that did not cause a color shift in cultures re-incubated in fresh medium.

## Results and discussion

**Figure 1:**
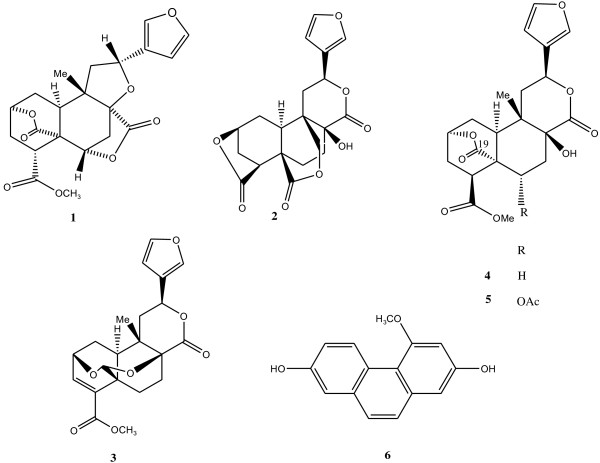
**Chemical structures of the compounds isolated from the bulbils of *Dioscorea bulbifera.***

The structural elucidation of the isolated compounds was achieved using physical and spectroscopic techniques as described in our research group. They were identified as diterpenoids bafoudiosbulbins A (**1**), B (**2**) [4], C (**3**) [5], F (**4**), G (**5**) [6] and 2,7-dihydroxy-4-methoxyphenanthrene (**6**) [16]. Their chemical structures are illustrated in Figure 1. These compounds together with the crude extract and fractions were tested for their antimicrobial activities and the results are reported in Tables 1 and 2.

The results of the MIC determinations (Table 1) indicated that when tested alone, the methanol crude extract, fractions DBB1 and DBB2 as well as compounds **2** to **5** were able to prevent the growth of all the fifteen studied microorganisms, including mycobacteria and Gram-negative bacteria, within the concentration range of 8 to 256 μg/mL. Compounds **1** and **6** showed selective activities, their inhibitory effects being noted respectively on 5/15 (33.3%) and 3/15 (20%) of the studied microorganisms. The lowest MIC value for the methanol extract and fractions (16 μg/mL) was obtained with DBB1 and DBB2 on *E*. *coli* AG100A and DBB2 on *M*. *tuberculosis* MTCS2. The lowest value for individual compounds (8 μg/mL) was recorded with compounds **3** on *M*. *smegmatis* and *M*. *tuberculosis* ATCC and MTCS2 strains. The activity of the samples on many MDR bacteria such as *E*. *aerogenes* EA289, CM64, *K*. *pneumoniae* KP63 and *P*. *aeruginosa* PA124 was better than that of chloramphenicol. When tested in the presence of the efflux pump inhibitor against Gram-negative bacteria expression active efflux (Table 1), the activity of most of the samples increased, the lowest MIC values obtained being 8 μg/mL for DBB1 and 2, compound **3** on *E*. *coli* AG100A. Results of MBC determinations (Table 2) also showed good activities for most of the tested samples. When tested in the absence of PAßN, MBC values not greater than 512 μg/mL were recorded on all studied microorganisms with fraction DBB2, compounds **2** to **5**, on 13/15 (86.7%) and 14/15 (93.3%) of the studied organisms respectively the crude extract and fraction DBB1. As previously observed with MIC values, compounds **1** and **6** showed poor activities in the MBC test. Similarly to MICs data, the MBC values of the samples decrease when they were associated with PAßN. Also, it can be noted (Figure 1) that significant reduction of the bacterial population is observed with the crude extract, fraction DBB2 and compound **3** at a concentration corresponding to their MBC values.

In the present work, broad spectrum of antimicrobial activities was recorded with the crude extract, fractions and compounds from *D*. *bulbifera*. Phytochemicals are routinely classified as antimicrobials on the basis of susceptibility tests that produce MIC in the range of 100 to 1000 mg/mL [17]. Activity is considered to be significant if MIC values are below 100 μg/mL for crude extract and moderate when 100<MIC<625 μg/mL [18,19]. Therefore, the activity recorded with the crude extract on the ATCC strain of *E*. *coli*, *E*. *aerogenes*, *K*. *pneumoniae*, *M*. *smegmatis* and *M*. *tuberculosis* as well as *E*. *coli* AG100A and *M*. *tuberculosis* MTCS2, can be considered as important. Similarly, the activity recorded with the two studied fractions from *D*. *bulbifera* on the majority of the studied microbial strains was also significant. The activity of compound **3** on three of the four tested mycobacterial strains can also be considered good. The MIC values obtained with compound **3** on MDR bacteria such as *E*. *aerogenes* EA289, CM64, *K*. *pneumoniae* KP63 and *P*. *aeruginosa* PA124 was lower than those of the reference drug, highlighting its good antibacterial potency.

**Figure 2:**
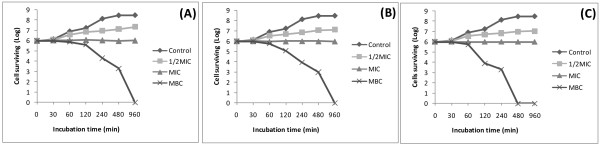
**Survival curve for *E.coli *ATCC8739 cells exposed to the crude extract** (**A**)**,fraction DBB2** (**B**) **and compound 3** (**C**)**.** (Control): MHB medium in DMSO 2.5% + inoculum.

Keen look of the MBC values of the tested samples, alone or in the presence of the efflux pumps inhibitor indicates that most of them are not more than fourfold their corresponding MICs. This proves that the killing effects of many tested samples could be expected on the sensitive strains [20]. This can also be confirmed by the reduction of cell survival at the MBC values of the crude extract, fraction DBB2 and compound **3** as observed in Figure 2. Enterobacteriaceae, including *K*. *pneumoniae*, *E*. *aerogenes* and *E*. *coli* as well as *P*. *aeruginosa* and *M*. *tuberculosis* have been classified as antimicrobial-resistant organisms of concern in healthcare facilities [21-25]. The good activities of the crude extract, fractions and compound **3** on most of the tested microorganisms belonging to MDR phenotypes such as *E*. *coli* AG102, *P*. *aeruginosa* PA124, *E*. *aerogenes* CM64, *K*. *pneumoniae* KP55 and KP63 as observed herein reinforces the hypothesis that *D*. *bulbifera* can be a potential source of antimicrobial drugs. It should be noted that active compounds from *D*. *bulbifera* are substrates of MDR bacteria efflux pumps, suggesting a possible use of an inhibitor in the fight against such strains. In this work, only compounds with inhibitory activity on *M*. *smegmatis* were tested on *M*. *tuberculosis*. However, it has been demonstrated that the sensitivity of *M*. *tuberculosis* is closer to that of *M*. *smegmatis*, a non pathogenic microorganism [26]. Therefore, this microorganism can be used for a preliminary study to select samples with potential activity against *M*. *tuberculosis*[26]. Hence, the results obtained herein are in accordance with such recommendation.

To the best of our knowledge, the antimicrobial activities of *D*. *Bulbifera* against MDR bacteria and mycobacteria strain as well as those of the isolated compounds is being reported for the first time. However the anti-salmonellal activity of compounds **1** and **2** was reported [4]. Also, a norditerpene, 8-epidiosbulbin E acetate, from *Dioscorea bulbifera* was found active against MDR *Escherichia coli* and *Pseudomonas aeruginosa* with MIC values of 200 and 400 μg/mL respectively [27]. The mechanism of the active compounds is still to be studied; nevertheless, membrane disruption could be suggested as one of the likely mechanisms of action of **1** to **5**, the compounds belonging to the terpenoids [28].

## Conclusion

The data reported herein are very important, taking in account the medical importance of the studied microorganisms. Hence, the overall results of the present investigation provided evidence that the crude extract *D*. *bulbifera* as well as some of the compounds, and mostly compounds **3** could be considered as potential antimicrobial drug.

## Competing interests

The authors declare that they have no competing interests.

## Authors’ contributions

VK, RBT and ATM carried out the study; VK, RBT and ATM wrote the manuscript; VK, LAT, LB, JJMM and NL supervised the work. All authors read and approved the final manuscript.

## Pre-publication history

The pre-publication history for this paper can be accessed here:

http://www.biomedcentral.com/1472-6882/12/228/prepub

## Supplementary Material

Additional file 1 **Table S1.** Gram-negative bacterial strains and features. The studied bacteria included reference ATCC strain of *E*. *coli* ATCC8739, *E*. *aerogenes* ATCC13048, *K*. *pneumoniae* ATCC12296 *and P*. *aeruginosa* PA01 as well as MDR strains *E*. *coli* AG100A and AG102, *E*. *aerogenes* EA-CM64 and EA289, *K*. *pneumoniae* Kp55 and Kp63, and *P*. *aeruginosa* PA124. Click here for file
