# Supplementary material for: Antibacterial activities of the extracts, fractions and compounds from Dioscorea bulbifera
Source: BMC Complement Altern Med. 2012 Nov 23;12:228. doi: 10.1186/1472-6882-12-228 (PMC3528471; doi:10.1186/1472-6882-12-228)
Supplement: Additional file 1 — Table S1. Gram-negative bacterial strains and features. The studied bacteria included reference ATCC strain of E. coli ATCC8739, E. aerogenes ATCC13048, K. pneumoniae ATCC12296 and P. aeruginosa PA01 as well as MDR strains E. coli AG100A and AG102, E. aerogenes EA-CM64 and EA289, K. pneumoniae Kp55 and Kp63, and P. aeruginosa PA124. [file 1472-6882-12-228-S1.doc]

**Antibacterial activities of the extracts, fractions and compounds from *Dioscorea bulbifera***

Victor Kuete^1,3,4*^, Rémy BetrandTeponno^2^, Armelle Tsafack Mbaveng^1^, Léon Azefack Tapondjou^2**^, Jacobus J. Marion Meyer^3^, Luciano Barboni^4^, and Namrita Lall^3^

*^1^ Department of Biochemistry, Faculty of Science, University of Dschang, Cameroon;*

*^2^ Department of Chemistry, Faculty of Science, University of Dschang, Cameroon;*

*^3^Department of Plant Science, Faculty of Agricultural and Biological Science, Pretoria 0002, South Africa;*

*^4^School of Science and Technology, Chemistry Division, University of Camerino, Via S. Agostino 1, I-62032 Camerino, Italy*

**Corresponding authors:**

**Tel : (237) 77 35 59 27 ; Fax: (237) 22 22 60 18. P.O. Box 67 Dschang, Cameroon; E-mail:* [*kuetevictor@yahoo.fr*](mailto:kuetevictor@yahoo.fr) *(Dr. V. Kuete)*

*** Tel.: +237 500 48 26; fax: +237 345 17 35. E-mail address: tapondjou2001@yahoo.fr (A.L. Tapondjou).*

***Authors e-mails:***

*Victor Kuete:* [*Kuetevictor@yahoo.fr*](mailto:Kuetevictor@yahoo.fr)*,*

*Rémy Betrand Teponno:* [*rteponno@yahoo.fr*](mailto:rteponno@yahoo.fr)

*Armelle Tsafack Mbaveng:* [*armkuete@yahoo.fr*](mailto:armkuete@yahoo.fr)

*Léon Azefack Tapondjou:* [*tapondjou2001@yahoo.fr*](mailto:tapondjou2001@yahoo.fr)

*Luciano Barboni:* [*luciano.barboni@unicam.it*](mailto:luciano.barboni@unicam.it)

*Jacobus J. Marion Meyer:* [*marion.meyer@up.ac.za*](mailto:marion.meyer@up.ac.za)

*Namrita Lall:* [*Namrita.Lall@up.ac.za*](mailto:Namrita.Lall@up.ac.za)

**Supporting information [see Main document]**

**Table S1.** Gram-negative bacterial strains and features

| Bacterial strains | Relevant features^a^ | References |
| --- | --- | --- |
|  |  |  |
| *E. coli* strains |  |  |
| ATCC8739 | Reference strains |  |
| AG100A | AG100 *ΔacrAB*::KAN^R^ | [1,2] |
| AG102 | AG100 over-expressing AcrAB pump | [3] |
| *E. aerogenes* |  |  |
| ATCC13048 | Reference strain |  |
| EA-CM64 | CHL^R^ resistant variant obtained from ATCC13048 over-expressing the AcrAB pump | [4] |
| EA289 | KAN sensitive derivative of EA27 | [4] |
| *K. pneumoniae* |  |  |
| ATCC12296 | Reference strain |  |
| Kp55 | Clinical MDR isolate, TET^R^ AMP^R^ ATM^R^ CEF^R^ |  |
| Kp63 | Clinical MDR isolate, TET^R^ CHL^R^ AMP^R^ ATM^R^ | [5] |
| *P. aeruginosa* |  |  |
| PA01 | Reference strain |  |
| PA124 | MDR clinical isolate | [4, 6] |

^a^AMP, ATM^R^, CEF, CHL^R^, KAN^R^, TET^R^. Resistance to ampicillin, aztreonam, cephalothin, chloramphenicol, kanamycin and tetracycline.

**Reference**

*1.* Okusu H D, Ma, Nikaido H: **AcrAB efflux pump plays a major role in the antibiotic resistance phenotype of *Escherichia coli* multiple-antibiotic-resistance Mar. mutants.** J Bacteriol 1996, **178:** 306-308.

*2.* Pradel E, Pagès JM: **The AcrAB-TolC Efflux Pump Contributes to Multidrug Resistance in the Nosocomial Pathogen *Enterobacter aerogenes.*** Antimicrob Agents Chemother 2002, **46:**2640-2643

*3.* Elkins CA, Mullis LB: **Substrate competition studies using whole-cell with the major tripartite multidrug efflux pumps of *Escherichia coli*.** Antimicrob Agents Chemother 2007,**51:**923-929.

*4.* Ghisalberti D, Masi M, Pagès JM, Chevalier J: **Chloramphenicol Chloramphenicol and expression of multidrug efflux pump in *Enterobacter aerogenes*.** Biochem Biophys Res Commun 2005, **328:**1113–1118.

*5.* Chevalier J, Pagès JM, Eyraud A, Malléa M: **Membrane permeability modifications are involved in antibiotic resistance in *Klebsiella pneumoniae.*** Biochem Biophys Res Commun 2000, **274:**496-499

*6.* Lorenzi V, Muselli A, Bernardini AF, Berti L, Pagès JM, Amaral L, Bolla JM : **Geraniol restores antibiotic activities against multidrug resistant isolate from gram-negative species.** Antimicrob Agents Chemother 2009, **53:** 2209-2211.
